# Selenium nanoparticles modulate histone methylation via lysine methyltransferase activity and *S*-adenosylhomocysteine depletion

**DOI:** 10.1016/j.redox.2023.102641

**Published:** 2023-02-23

**Authors:** Benoit Toubhans, Nour Alkafri, Marcos Quintela, David W. James, Caroline Bissardon, Salvatore Gazze, Franziska Knodel, Olivier Proux, Alexandra T. Gourlan, Philipp Rathert, Sylvain Bohic, Deyarina Gonzalez, Lewis W. Francis, Laurent Charlet, R. Steven Conlan

**Affiliations:** a Swansea University Medical School, Swansea University, Swansea, SA2 8PP, UK; b Université Grenoble Alpes, ISTerre, 38000, Grenoble, France; c Université Grenoble Alpes, INSERM, UA7 STROBE, Synchrotron Radiation for Biomedicine, Grenoble, France; d Department of Biochemistry, Institute of Biochemistry and Technical Biochemistry, University of Stuttgart, D-70550, Stuttgart, Germany; e OSUG, UAR 832 CNRS, Université Grenoble Alpes, 38041, Grenoble, France; f ESRF, European Synchrotron Radiation Facility, CS, 40220, 38043, Grenoble, Cedex 9, France

## Abstract

At physiological levels, the trace element selenium plays a key role in redox reactions through the incorporation of selenocysteine in antioxidant enzymes. Selenium has also been evaluated as a potential anti-cancer agent, where selenium nanoparticles have proven effective, and are well tolerated *in vivo* at doses that are toxic as soluble Se. The use of such nanoparticles, coated with either serum albumin or the naturally occurring alkaline polysaccharide chitosan, also serves to enhance biocompatibility and bioavailability. Here we demonstrate a novel role for selenium in regulating histone methylation in ovarian cancer cell models treated with inorganic selenium nanoparticles coated with serum albumin or chitosan. As well as inducing thioredoxin reductase expression, ROS activity and cancer cell cytotoxicity, coated nanoparticles caused significant increases in histone methylation. Specifically, selenium nanoparticles triggered an increase in the methylation of histone 3 at lysines K9 and K27, histone marks involved in both the activation and repression of gene expression, thus suggesting a fundamental role for selenium in these epigenetic processes. This direct function was confirmed using chemical inhibitors of the histone lysine methyltransferases EZH2 (H3K27) and G9a/EHMT2 (H3K9), both of which blocked the effect of selenium on histone methylation. This novel role for selenium supports a distinct function in histone methylation that occurs due to a decrease in *S*-adenosylhomocysteine, an endogenous inhibitor of lysine methyltransferases, the metabolic product of methyl-group transfer from *S*-adenosylmethionine in the one-carbon metabolism pathway. These observations provide important new insights into the action of selenium nanoparticles. It is now important to consider both the classic antioxidant and novel histone methylation effects of this key redox element in its development in cancer therapy and other applications.

## 1 Introduction

Selenium compounds contribute to the maintenance and integrity of cellular systems by influencing cellular redox states and the capacity to detoxify compounds, free radicals and reactive oxygen species [1]. Thioredoxin reductases, for example, which contain selenocysteine, are present in the cytosol (TrxR1) and mitochondria (TrxR2), are involved in the reduction of oxidized thioredoxins, can catalyse NADPH, control ascorbate levels, and regulate metabolism [2]. Selenium is also involved in the biosynthesis of diverse molecular components that are required for important cellular functions including deoxyribonucleoside triphosphates for DNA, and the reduction of oxidized proteins, as well as having roles in diverse regulatory mechanisms such as redox, apoptosis, immunomodulation and the formation of methyl donor compounds [3].

Selenium has been investigated as a potential anti-cancer agent for the reduction of the risk of different malignancies including colorectal, lung, and prostate cancer, as well as an adjuvant to conventional anti-tumour therapies [4]. Such use is limited, however, by the narrow therapeutic window of selenium, since exceeding the daily recommended dose of 60–70 μg can lead to serious illness such as selenosis that occurs at doses above 400 μg [4,5]. Nanoparticle formulations of selenium, on the other hand, have proven to be more tolerated *in vivo* than aqueous/organic selenium. Studies have show that the toxicity of selenomethionine is higher than that of Se nanoparticles (SeNPs) as indicated by acute liver injury, and short-term toxicity, where (SeNPs) caused 10% mortality at a dose of 36 mg Se/kg whereas selenomethionine caused 90% mortality at a dose of 32 mg Se/kg [6,7]. Similarly for selenite *in vivo* LD50 acute toxicity has been reported as 15.7 mg Se/kg body weight, whereas with (SeNPs) acute toxicity was 7-fold less at 113.0 mg Se/kg body weight following a single dose treatment and monitoring over 14 days [8]. When administered at 6 mg/kg daily for 12 consecutive days (SeNPs) were less toxic than selenite showing less suppression of growth, moderate redox stress, and lower levels of the liver function enzymes ALT and AST [9].

The anti-proliferative effect of selenium is achieved through different mechanisms of action including acting as pro-oxidants, generating ROS, boosting cellular antioxidant defences, inducing apoptosis, influencing cell signalling and autophagy, interfering with protein-kinase signalling, and inducing cell cycle arrest^4^. These mechanisms include the regulation of protein kinase signalling, caspase activation, and phosphorylation of p53 [10]. In-vitro SeNPs have been shown to increase the effect of irradiation in MCF-7 breast cancer cells due to cell cycle arrest, the induction of autophagy and the production of ROS [10]. Additionally, significant anti-proliferative activity against cancer cells has been observed using paclitaxel-loaded SeNPs, revealing disruption of mitochondrial membrane potential orchestrated with the induction of ROS, and leading to the activation of caspase induced apoptotic cell death [11].

Here we aimed to determine the effect of exposure to SeNPs, coated with either serum albumin or the chitosan to enhance biocompatibility and bioavailability, in two pathologically distinct ovarian cancer cell models on redox response and cell viability [12,13]. We sought to determine the mechanism of action resulting in decreased cell viability, and directed by RNA sequencing data, investigated the impact of SeNP treatment on the regulation of histone methylation. Having observed that SeNP activity was inhibited by specific histone lysine methyltransferase inhibitors we explored the underlying one carbon cycle pathway responsible for this effect. Together these investigations uncovered a novel epigenetic regulatory mechanism altered following SeNP exposure.

## 2 Methods

### 2.1 Nanoparticles

BSA and chitosan-coated SeNPs were purchased from NANOCS (New York, USA) supplied at a fixed concentration 2 mg/ml, with a manufacturer-defined diameter of 30–100 nm for each type of nanoparticle (Supplementary Fig. S2). Size shape and charge analysis was conducted by Dynamic Light Scattering and Zeta Potential measurement using a ZetaSizer Nano (Malvern Instruments, Malvern, UK) with a 173° scattering angle using SeNPs at 1 μg/mL in water (reflective index of 1.33) at 25 °C.

### 2.2 Spheroid growth, treatment, and viability assay

Five thousand cells/well were plated in 96-well Ultra Low Attachment coated round bottom plates (Corning, UK). After spheroid formation (usually after 24 h), 100 μL of fresh medium containing a 2X concentration of sodium selenite (Na_2_SeO_3_) or SeNPs (BSA or chitosan coated) were added. Cell viability was determined using a CellTiterGlo assay (Promega, UK). For the viability assay, an increasing dose range (0.01 μg/mL to 20 μg/mL selenite of SeNPs) was applied by dilution in the appropriate medium for 24, 48 or 72 h. After the treatment, 100 μL of media was removed from wells and 100 μL of CellTiterGlo added. Plates were shaken for 5 min and equilibrated at room temperature for 25 min before luminescence measurements were taken (BMG Labtech Fluostar Omega, UK). IC20 and IC50 doses were determined as the concentration required to reduce the luminescence signal by 20/50%. The IC20/50 values shown are the result of a minimum of five independent experiments performed with 4 technical repeats. IC50 values calculated as 1.25 μg/mL for SeNP-BSA and 3 μg/mL for SeNP-chitosan, which were only slightly less than that for selenite (IC50 0.6 μg/mL) in SKOV-3 spheroids. Whereas in OVCAR-3 models the IC50 was calculated as 5 μg/mL for both SeNP-BSA and SeNP-chitosan compared to selenite (IC50 10 μg/mL). IC20 values calculated as 0.15 μg/mL for SeNP-BSA and 0.15 μg/mL for SeNP-chitosan, which were slightly lower than that for selenite (IC20 0.3 μg/mL) in SKOV-3 spheroids. Whereas in OVCAR-3 models the IC20 was calculated as 0.6 μg/mL for both SeNP-BSA and SeNP-chitosan compared to selenite (IC20 0.3 μg/mL).

### 2.3 Cell culture

OVCAR-3 ovarian cancer cells (ATCC, Maryland, US) were cultured in RPMI-1640 medium (Sigma-Aldrich, UK) supplemented with 20% Fetal Bovine Serum (FBS, Sigma-Aldrich), 5 μg/mL insulin (Sigma-Aldrich), and 1% penicillin-streptomycin (v/v) solution (Sigma-Aldrich). SKOV-3 ovarian cancer cells (ATCC, Maryland, US) were cultured in McCoy's 5A medium (Sigma-Aldrich) supplemented with 10% Fetal Bovine Serum (FBS, Sigma-Aldrich, UK), and 1% penicillin-streptomycin (v/v) (Sigma-Aldrich, UK). Cells were maintained at 37 °C and 5% CO_2_ and routinely passaged using 0.25% trypsin-0.1%EDTA (v/v).

### 2.4 Cell growth and Se treatment

SKOV-3 and OVCAR-3 cells were cultured in growth medium to 80%confluency on sterile plastic petri dishes (Corning, UK) at 37 °C and 5% CO2. After 48 h incubation culture medium was removed and 2 mL of fresh medium containing aqueous Se (Na_2_SeO_3_) or SeNPs. SKOV-3 cells were treated with IC20; selenite 3 μg/mL, BSA-SeNP 6 μg/mL or chitosan-SeNP 13 μg/mL. OVCAR-3 cells were treated with selenite 40 μg/mL, BSA-SeNP 20 μg/mL or chitosan-SeNP 18 μg/mL. Following addition of selenium treatment cells were incubated for a further 48 h prior to analysis. A minimum of three biological repeats were conducted for each cell type and treatment.

### 2.5 SDS–PAGE and protein blotting

For cell lysis, the media was aspirated from cells, which were washed twice in ice-cold phosphate-buffered saline (PBS; Fisher Scientific). Cells were lysed in ice-cold RIPA buffer (150 mM NaCl, 1% Triton X-100, 0.5% sodium deoxycholate, 0.1% SDS, 50 mM Tris pH 8.0) supplemented with a Halt™ Protease Inhibitor Cocktail (100X) (78430, Fisher Scientific). Cells in lysis buffer were agitated on a shaker for 40 min at 4 °C, and the removed lysates were cleared by centrifugation at 21,500×*g* for 10 min at 4 °C. The supernatant was transferred into new microfuge tubes and stored at −20 °C until required.

For equal loading of samples in western blots, the bicinchoninic acid (BCA) protein assay was employed to quantify the protein concentrations in samples. 30 μg of total protein from each cell lysate was used.

Cell lysates were subjected to sodium dodecyl sulphate polyacrylamide gel electrophoresis (SDS-PAGE). The separated proteins were transferred by a wet transfer method onto an activated polyvinylidene fluoride membrane (Millipore, UK). Membranes were incubated for 1 h at room temperature in a blocking buffer, which was either Tris-buffered saline-Tween 0.1% (TBS-T; Fisher Scientific, UK) containing 5% bovine serum albumin (BSA; Fisher Scientific, UK) if probing for phosphorylated proteins. After blocking, membranes were incubated with primary antibodies diluted in an appropriate blocking buffer overnight at 4 °C, washed (3 × 5 min) with TBS-T, and then incubated with appropriate horseradish peroxidase (HRP)-conjugated secondary antibody diluted in an appropriate blocking buffer for 2 h at room temperature. Following washing with TBS-T (3 × 5min), membranes were incubated with an enhanced chemiluminescence (ECL) development reagent (Clarity Western ECL substrate, Biorad, UK) for 3 min and visualised by (ChemiDoc XRS, BioRad, UK), and band intensities quantified using ImageJ software normalising expression to GAPDH.

Primary antibodies recognising human proteins used were: Caspase 3: rabbit polyclonal 9662 (Cell Signalling Technology (CST), UK), ATG5: rabbit polyclonal 2630, (CST), H3K4me3: rabbit polyclonal PA517420 (Thermo, UK), H3K27me3: rabbit polyclonal PA531817 (Thermo, UK), H3K9me2: rabbit polyclonal (CST), H3 (1B1B2): mouse monoclonal 14269 (CST) and GAPDH mouse monoclonal sc-47724 (Santa Cruz, UK), Bcl-2 rabbit polyclonal ab196495 (Abcam, UK), p21(CDKN1A): rabbit monoclonal 2947 (CST) and β-actin rabbit polyclonal 4967 (CST) were used at a concentration of 200 μg/ml overnight at 4 °C. Blots were washed and then incubated with the appropriate secondary antibodies (goat anti-mouse ab150113 (Abcam, UK) or goat anti-rabbit ab6721 (Abcam, UK HRP secondary) at a concentration of 400 μg/ml. Immuno-reacting proteins were visualised (ChemiDoc XRS, BioRad, UK), and band intensities were quantified using ImageLab software normalising expression to GAPDH.

### 2.6 ROS assay

SKOV-3 and OVCAR-3 cells were seeded as monolayers at 20,000 cells per well in a black 96-well plate and cultured overnight. Following removal of media, cells were washed once with PBS, then incubated for 1 h with the Cellular Reactive Oxygen Species Detection reagent (Red Fluorescence, Abcam, UK, 186027). An IC20 concentration of selenite or SeNPs (mentioned previously) was then added and the plate was incubated at 37 °C for the duration of the assay. Fluorescence was analysed at different time points from 30 min to 10 h (excitation filter 520 nm, emission filter 605 nm, BMG Labtech Fluostar Omega, UK).

### 2.7 RT-qPCR

Cellular total RNA was isolated using RNeasy Mini Kit (Qiagen, Germany) according to the manufacturer's protocol and purity and concentration were determined using a spectrophotometer (ND-1000; NanoDrop Technologies, USA). Following RNA extraction and quantification, cDNA synthesis was carried out following the manufacturer's recommendations, using the RETROscript® kit two-step method (Invitrogen Ltd., UK). Following cDNA synthesis from 100 ng of RNA, each sample was analysed by qPCR in triplicate using iQ SYBR Green supermix (BioRad, UK) and gene-specific primers (Sigma-Aldrich, UK) to evaluate differential gene expression GAPDH (GAPDH Forward: GTCCACTGGCGTCTTCAC, Reverse: CTTGAGGCTGTTGTCATACTTC) and TrxR1 (Forward: TCCCAAGTCCTATGACTATGACC, Reverse: CCATATTGGGCTGCCTCCTT). Serial dilutions of cDNA were used to plot a calibration curve, and gene expression was quantified by plotting threshold cycle values. Expression levels were normalized to values obtained for the reference gene (GAPDH) and relative expression expressed as the mean fold induction ± standard deviation. Statistical differences between the treatment groups and the control were determined by analysis of variance (ANOVA) (where p < 0.05 was considered significant).

### 2.8 RNA-sequencing

For each condition, a total of 96 independently cultured spheroids were pooled. Extracted RNA from pooled samples underwent quality control assessment using the RNA Tapestation 2200 (Agilent). cDNA libraries were prepared using the SENSE mRNA-Seq Library Prep Kit V2 (Lexogen) prior to RNA-sequencing (RNA-Seq, genomic platform, Ecole Normale Supérieure de Lyon). Raw Fastq files were quality-checked using FastQC, a quality-control tool for high throughput sequencing data, prior to alignment to the hg38 indexed transcriptome using Bowtie2 [14]. The eXpress software [15] was used to quantify expression from the transcriptome mapping and derive count data and the differential expression tool package DESeq2 [16], implemented within R, was used to correct for multiple hypothesis testing and determine significantly modified transcripts (FDR <0.1) (Supplementary 1). Raw and processed RNA-Seq data is deposited in the GEO Dataset with accession number GSE149397. All major PANTHER terms were tested for over-representation (GO-Biological Processes or Reactome, e.g. binomial) and gene-set enrichment by comparing the lists of genes expressed in different experimental conditions. The results are displayed showing the differential distribution of significantly enriched clusters of genes compared to the overall expression tendency within samples. The Database for Annotation, Visualization and Integrated Discovery (DAVID; https://david.ncifcrf.gov/) was used to provide functional annotation of gene lists and results displayed with Fisher's Exact test used to measure the gene-enrichment in annotation terms.

### 2.9 Epigenetic probes

Epigenetic probes were supplied by the Structural Genomics Consortium (SGC) under an Open Science Trust Agreement: https://www.thesgc.org/click-trust. Probes were diluted in DMSO to a final concentration of 20 μM. UNC1999 and GSK343 inhibitors and an inactive control probe UNC2400 were used to evaluate EZH1/2. The inhibitor MRK-740 and inactive control probe MRK-740 N were used for PRDM9. UNC0642 and A-366 were used to evaluate G9a/EHMT2. Cells were treated for 1–3 days with different probes to reach IC90 of the targeted methyltransferase. Cells were then treated for 48 h with selenite or SeNP at sub-lethal doses, protein was extracted and probed using antibodies targeting specific histone modifications as described above.

### 2.10 Transmission electron microscopy (TEM)

Transmission electron microscopy (TEM) sections were prepared as previously described [17]. Briefly, spheroids were pelleted and vitrified by high-pressure freezing (HPM100, Leica Microsystems) to −90 °C for 80 h in acetone with 1% OsO_4_. The temperature was then raised from 1 °C/h to 30 °C and samples were rinsed 4 times in acetone. Samples were infiltrated with agar low viscosity resin (LVR, Agar scientific) in acetone for 3 h. After polymerisation for 24 h at 60 °C, 70–400 nm sections were obtained using an ultra-microtome (UC7, Leica Microsystems). Sections were collected on formvar-carbon-coated 100 mesh copper grids and post-stained for 10 min with 2% aqueous uranyl acetate, rinsed and incubated for 5 min with lead citrate. Grids were analysed using a Tecnai 12 FEI Microscope (120 kV) at different magnifications. 200-mesh formvar and carbon-coated copper grids (Agar Scientific) were incubated for 60 min with a 50 μl drop of the relevant SeNP solution. Grids were washed in ultrapure water for 5 min, three times, and stained with 2% phosphotungstic acid (Agar Scientific) in water, pH 7, for 10 min. Excess solution was absorbed with filter paper and grids were allowed to air-dry overnight. Grids were imaged on a JEM-1400 Flash Transmission Electron Microscope at 120 kV.

### 2.11 High energy resolution fluorescence detected X-ray absorption spectroscopy

Energy Resolution Fluorescence Detected X-ray Absorption Spectroscopy (HERFD-XAS) was used to determine Se speciation. Measurements were performed on FAME-UHD beamline at the European Synchrotron Radiation Facility (ESRF, Grenoble, France) at the selenium K-edge (12658 eV). The samples were analysed at 10 K to avoid radiation damages using a crystal analyzer spectrometer (CAS) equipped with 6 Ge(844) bent crystals (1 m radius of curvature), allowing an energy bandwidth for the fluorescence detection around 3.3eV. Data analysis was performed by linear combination fitting of the so-obtained spectra using a set of model compounds spectra previously measured obtained on the same conditions [18].

### 2.12 SAH ELISA

Cells were treated for 24 h with SeNP or selenite with (mentioned in 2.4) (+) or without AHCY inhibitor (3-deazaneplanocin A) at 1 μM. Cells were harvested by centrifuging at 2000×*g* for 10 min at 4 °C and the cell pellet was homogenized on ice in 1–2 mL cold PBS. This homogenized suspension was centrifuge at 10,000×*g* for 15 min at 4 °C, and the supernatant was removed and stored at −80 °C prior to measurement. Quantification of intracellular SAH concentration was conducted using an *S*-Adenosylmethionine (SAM) and *S*-Adenosylhomocysteine (SAH) ELISA Combo Kit (STA-671-C; from Cell Biolabs, INC. (catalogue #STA-671-C); Generon; Berkshire, UK) following the manufacturer's protocol.

### 2.13 AHCY activity assay

AHCY activity assay was performed in a 96-well plate using an AHCY activity assay kit (Fluorometric) (Abcam, Cambridge, UK) that allows quantitative measurement of homocysteine concentration in cell extracts, according to the manufacturer's instructions. The concentration of homocysteine in cell extracts was used as a readout for in vitro AHCY activity, as the AHCY enzyme hydrolyses SAH to homocysteine and adenosine. The fluorescence of the samples was measured at Ex/Em = 535/587 nm in 5 min after mixing and 30 min after incubation using (BMG Labtech Fluostar Omega, UK). A serial dilution of adenosine was included in each assay to obtain a standard curve. AHCY activity was calculated using the adenosine standard curve according to the manufacturer's instructions.

### 2.14 ChIP-seq analysis

ChIP-Seq datasets were downloaded from repository GSE118406 [19] using prefetch and fasterq-dump functions from the SRA toolkit. Sequence reads were mapped against human reference genome build hg38 using Bowtie2 [20]. Peak calling was performed using MACS2 with default settings [21].

### 2.15 Statistical analysis

All data presented are from a minimum of three biological repeats, with technical repeats included per sample, as denoted. Data normality was analysed using the Kolmogorov Smirnov test, with normally distributed data analysed with the one-way and two-way analysis of variance (ANOVA) and the non-parametric data analysed using Kruksal Wallis followed by the Mann–Whitney U pairwise test. In all cases in which ANOVA was significant, multiple comparison methods were used. Differences were considered significant for (*P ≤ 0.05, **P ≤ 0.01, ***P ≤ 0.001, ****P ≤ 0.0001 and ns for non-significant). All data were analysed in Mini Tab 14.

## 3 Results

### 3.1 Selenium nanoparticles effective against 3D ovarian cancer models

**Fig. 1:**
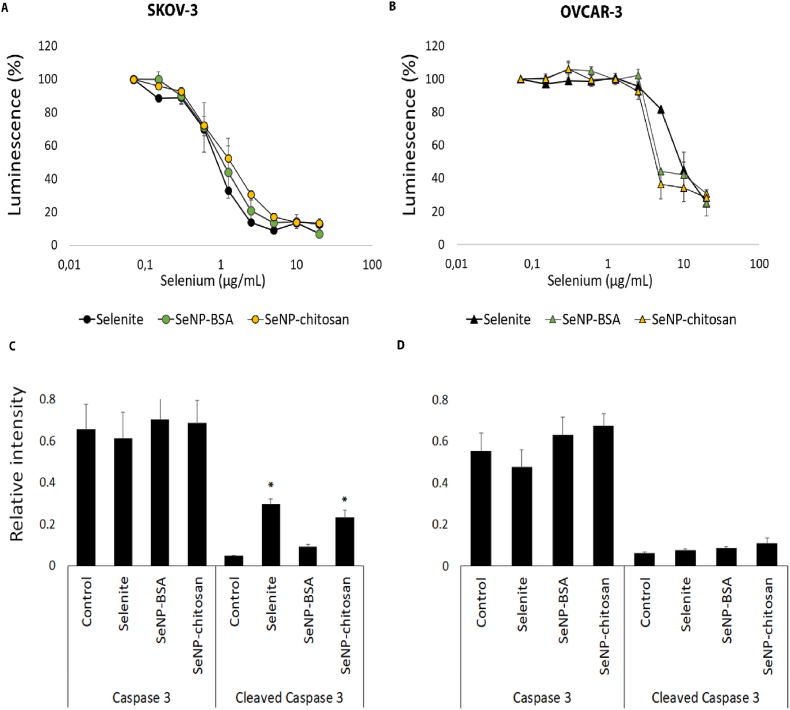
Ovarian cancer cell cytotoxicity in the presence of SeNP formulations. SKOV-3 (A) and OVCAR-3 (B) were grown as 5 × 10^3^ cell spheroids for 24 h and then treated with an increasing concentration range (0–20 μg/mL) of selenite, BSA-SeNP or chitosan-SeNPs for 24 h and cellular cytotoxicity monitored. Cytotoxicity was evaluated by a CellTiterGlo endpoint assay. OVCAR-3 cells were more resistant to selenium treatment than SKOV-3 cells. Mean ( ± SD) relative to control luminescence values are shown from five independent experiments. In SKOV-3 cells relative caspase-3 levels (C) were similar between control and selenium treated levels, 0.65 control, 0.68 selenite, 0.70 SeNP-BSA and 0.68 SeNP-chitosan. Selenite and SeNP-chitosan treatments caused significant levels (p < 0.05) of caspase-3 cleavage with levels of 0.29 and 0.23 detected respectively. The only moderate increase in caspase 3 cleavage was observed for SeNP-BSA treatment (0.09). In OVCAR-3 no changes in caspase 3 (D) were seen for selenite (0.47), SeNP-BSA (0.62) and SeNP-chitosan (0.67) in comparison with control (0.55), and cleaved caspase levels were very low for each condition. The data represents the mean ± SD of three individual experiments.

Previously we demonstrated that the direct application of SeNPs to cell monolayers had a cytotoxic effect [13]. However, to better recapitulate an *in vivo* solid tumour setting we evaluated their effect on 3D ovarian cancer models. Two distinct cell lines, OVCAR-3 and SKOV-3 were used to create spheroids and SeNPs IC50 values determined (Fig. 1A and B). BSA (108 ± 30 nm; 0.123 ± 0.002 and −51.2 ± 15.8 mV) and chitosan (320 ± 221 nm; 0.220 ± 0.012 and 16.4 ± 4.4 mV) coated SeNPs were first analysed using standard biophysical techniques (see methods section) for size, uniformity (PdI) and zeta potential and displayed characteristics consistent with previous reports [13] (Supplementary Fig. S1). Both BSA and chitosan coated nanoparticles caused significant reductions in cell viability following treatment of spheroids, with IC50 values calculated as 1.25 μg/mL for SeNP-BSA and 3 μg/mL for SeNP-chitosan, which were only slightly less than that for selenite (IC50 0.6 μg/mL) in SKOV-3 spheroids (Fig. 1A). In OVCAR-3 models the IC50 was calculated as 5 μg/mL for both SeNP-BSA and SeNP-chitosan compared to selenite (IC50 10 μg/mL) (Fig. 1B). Further analysis revealed that exposure of SKOV-3 spheroids to SeNP-chitosan as well as selenite resulted in significantly increased (p < 0.05) caspase-3 cleavage levels, whereas only a moderate increase in caspase-3 cleavage was observed following SeNP-BSA treatment (Fig. 1C). In contrast there was no change in caspase-3 levels following selenium treatments in OVCAR-3 cells.

**Fig. 2:**
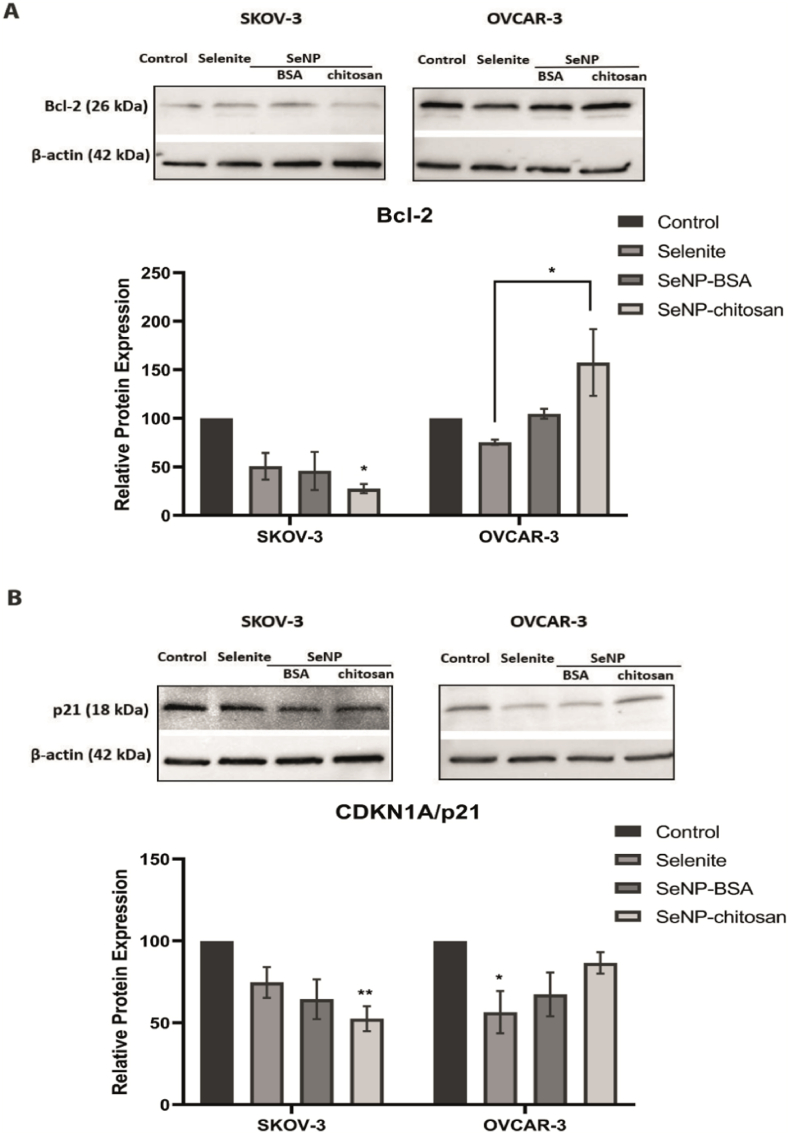
SKOV-3 and OVCAR-3 cells were treated for 24 h with SeNP or selenite. In SKOV-3 SeNP-chitosan treatment decreased significantly (a) Bcl-2 and (b) CDKN1A levels compared to OVCAR-3 where no significant decrease was observed. In SKOV-3 cells the IC20 concentrations used; selenite 3 μg/mL, BSA-SeNP 6 μg/mL and chitosan-SeNP 13 μg/mL, and in OVCAR-3 cells concentrations; selenite 40 μg/mL, BSA-SeNP 20 μg/mL and for chitosan-SeNP 18 μg/mL. Data are mean ± SEM; one-way ANOVA with Tukey multiple comparisons post-hoc analysis; **p < 0.01, *p < 0.05 and ns, not significant vs respective Control (untreated) for each treatment (n = 3 independent experiments).

To better understand the observed mechanistic differences of these responses to SeNPs we assessed the expression of two apoptotic markers Bcl-2 and p21(CDKN1A). SKOV-3 cells showed a decrease in levels of both proteins, which was significant following SeNP-chitosan treatment (p < 0.05 for Bcl-2 and p < 0.01 for p21). For OVCAR-3 cells there was no significant decrease in either marker following nanoparticle treatment (p > 0.05; Fig. 2). It appears therefore that SeNPs trigger death via apoptosis in the SKOV-3 spheroids, whereas loss of OVCAR-3 spheroid viability is not apoptosis related.

### 3.2 SeNP penetration and uptake in 3D ovarian cancer cell models

**Fig. 3:**
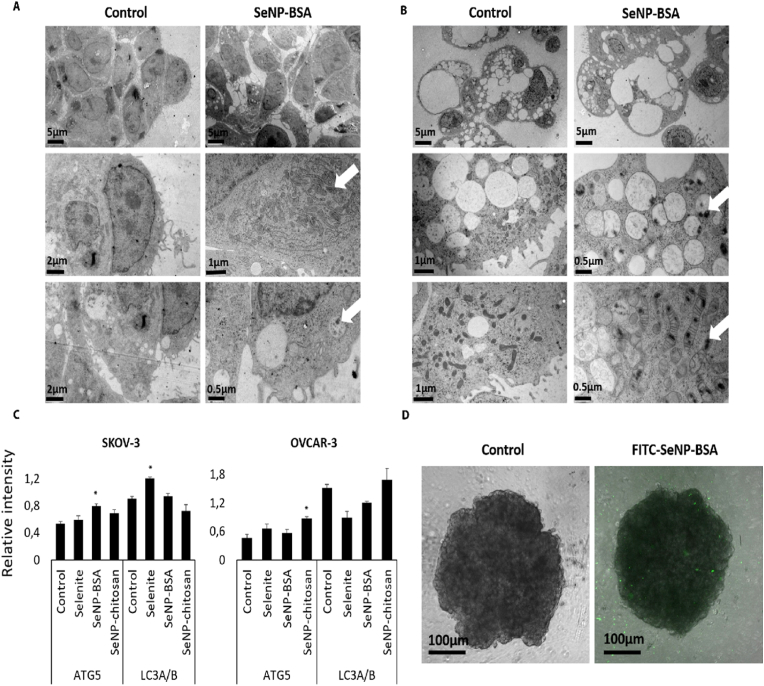
SeNP accumulation in SKOV-3 and OVCAR-3 spheroids. SeNPs penetrate and accumulate in SKOV-3 and OVCAR-3 spheroids. SKOV-3 (a) and OVCAR-3 (b) cell spheroids were treated with BSA-SeNPs at IC20 concentrations for 24 h and imaged by TEM. Scale bars are displayed on the images (between 0.5 and 5 μm). SeNP accumulation was observed in vesicles and mitochondria. SKOV-3 show a limited accumulation of SeNPs. All images are representative of a minimum of 3 biological repeats. SKOV-3 and OVCAR-3 cell spheroids were treated for 24 h with selenite or SeNPs and profiling autophagy markers (c). Control and selenite treated SKOV-3 displayed similar levels of ATG5 (respectively 0.53 and 0.59), whereas SeNP-BSA treated cells showed a significant increase (0.79, p = 0.05) of ATG5 levels. SeNP-chitosan treated SKOV-3 cells showed a non-significant elevated level of ATG5 (0.68). Selenite, but not SeNPs, had significantly increased LC3B levels in SKOV-3 cells. OVCAR-3 cells displayed an increase of ATG5 expression in the different conditions (selenite 0.65, SeNP-BSA 0.56, SeNP-chitosan 0.87) compared to the control (0.46) that was only significant for SeNP-chitosan (p < 0.05). Data represent the mean ± SD of three biological replicates *p < 0.05 significant vs respective Control (untreated) for each treatment. To determine nanoparticle penetration, SKOV-3 cells were treated with FITC-tagged-SeNP-BSA for 24 h (d). Confocal microscope (Ex 495 nm/Em 521 nm) imaging shows 50 μm z-stacks of a 300 μm diameter spheroid (scale bare 100 μm). Local fluorescence was observed inside the spheroid demonstrating nanoparticle penetration.

Having demonstrated the efficacy of SeNPs on cell viability we sought to establish the fate of the particles. Initial analysis was undertaken to determine the location of SeNPs, and we observed that they were able to penetrate at least 80 μm into 3D spheroid tumour models. 30 nm electron dense particles corresponding to SeNP aggregates were observed by TEM in vacuoles and mitochondria in cells treated with SeNP-BSA (Fig. 3A and B) or SeNP-chitosan (Supplementary Fig. S2) and confirmed using FITC-tagged SeNPs (Fig. 3). To assess whether these vacuolar structures were autophagosomes, the expression of autophagy markers was measured [22] and ATG5 levels were found to be up-regulated by SeNP-BSA, but unaffected by selenite or SeNP-chitosan in SKOV-3 spheroids (Fig. 3C), whereas there was an increase of LC3B with selenite, but not with either SeNP types (Fig. 3C). In OVCAR-3 spheroids, ATG5 expression increased following all SeNP-chitosan, consistent with these cells being more resistant to selenium, and with constitutively activated autophagy [23].

### 3.3 Evaluation of Se speciation and ROS response of SeNPs in ovarian cancer models

**Fig. 4:**
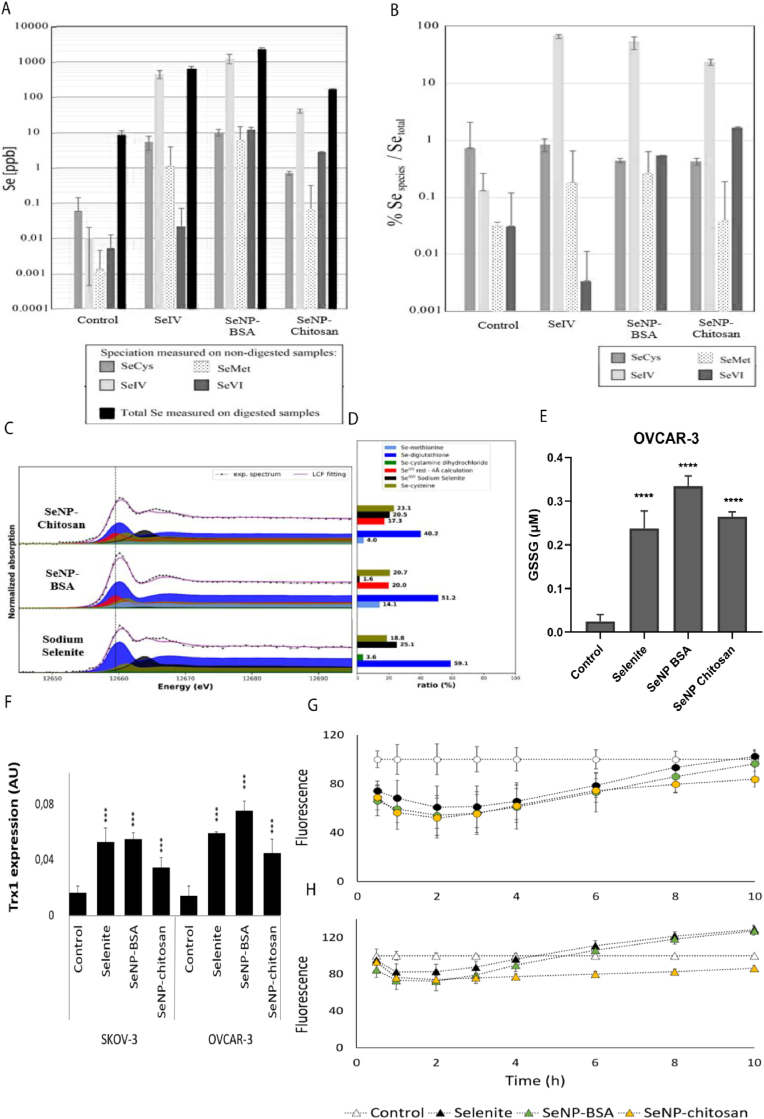
Evaluation of different Se-species and total Se concentration using HPLC-ICP-QQQ-MS on (A) OVCAR-3 cell supernatants after 24 h treatment with SeNPs or sodium selenite, and (B) as a percentage of Se-chemical species normalized by the total Se quantity. SeCys: Selenocysteine, SeMet: Selenomethionine, SeIV: Se(IV) and SeVI: Se(VI). Evaluation of selenium speciation using synchrotron high energy resolution fluorescence detected X-ray absorption spectroscopy (HERFD-XAS) on OVCAR cells after 24 h treatment SeNP and sodium selenite treated at IC20. (C) Linear combination fitting (LCF) of sample by reference selenium species (D) selenium species ratios present in cell samples (Error bar indicates 5% on these proportions). Selenium 0 red was calculated to obtain a reference spectrum that was used in the fit of all samples Measurements were made in duplicate on two different samples. Quantification of GSSG (E) Cells were treated with SeNPs for 24 h before the addition of a GSH blocking agent to prevent GSH reduction and subsequently treated with a reducing agent to convert GSSG/GS-Se-SG to GSH prior to quantification via GSH-dependent conversion of luciferin-NT, a GSH probe, to luciferin by a glutathione-*S*-transferase and showed an increase in GSSG concentrations in OVCAR-3 cells after SeNP treatment. Quantitation was made using a standard curve constructed with 0–16 μM of GSH. The limit of detection was 0.5 nM GSSG The data represents the mean ± SD. Effect of SeNPs and selenite on TrxR1 selenium-related gene expression and ROS production in OVCAR-3 and SKOV-3. (F) TrxR1 expression increased significantly (p < 0.001) TrxR1 RNA levels in SKOV-3 (Relative value of 0.01 control, 0.05 selenite and SeNP-BSA, 0.03 SeNP-chitosan) and OVCAR-3 (Relative value of 0.01 control, 0.06 selenite, 0.07 SeNP-BSA and 0.04 SeNP-chitosan) after 24 h of treatment with SeNPs or selenite. SKOV-3 (G) and OVCAR-3 (H) cells were incubated with ROS probes and treated with IC20 concentrations of SeNPs or selenite. ROS red fluorescent assay results showed that all treatments caused a decrease the production of ROS in SKOV-3 cells. In OVCAR-3 cells, following SeNP-BSA and selenite exposures ROS production peaked above control levels after 6h. The data represents the mean ± SD of three individual experiments. (For interpretation of the references to color in this figure legend, the reader is referred to the Web version of this article.)

Insoluble elemental selenium 0 (Se(0)), the main species of Se in SeNPs, elicited a number of similar cellular and molecular responses to aqueous selenite. We therefore sought to understand whether the Se(0) in SeNPs underwent biotransformation to soluble forms of Se upon exposure to ovarian cancer cells and thus account for the similar bioactivity as selenite. Using a combination of HLPC-ICP-MS and synchrotron high energy resolution fluorescence detected X-ray absorption spectroscopy (HERFD-XAS) OVCAR-3 cells were analysed 24 h after treatment with SeNP to evaluate selenium speciation and form (Fig. 4A and B). We found that whilst Se(0) remained present within the cells, the main selenium species was Se(IV), suggesting that SeNPs undergo conversion to form similar species as aqueous selenite, and thus would follow the same biological fate. Furthermore HERFD-XAS identified a 40–60 fold increase in the ratio of selenodigluthatione (GS-Se-SG) compared to untreated cells using reference spectra of compounds representative of the common selenium forms and a linear combination fit [18,24]. Other chemical species that were detected included alkyl selenide as selenocysteine, where an 8–23 fold increase in ratio was observed following only SeNP treatment (Fig. 4C and D).

The introduction of Se into a biological system can result in the increase of both glutathione disulfide GSSG and selenodiglutathione GS-Se-SG from glutathione GSH and SeO3(^2-^), and consequently, can both be reduced to GSH [25,26]. We assessed the accumulation of GSSG/GS-Se-SG using a direct luminescence assay. Increases in GSSG/GS-Se-SG ratios were observed in response to SeNP treatment in both OVCAR-3 (Fig. 4E) and SKOV-3 cells (Supplementary Fig. S3). SeNP treatment also resulted in an anticipated increase in thioredoxin reductase 1 (TrxR1) expression, which catalyses the conversion of SeO3(^2-^) to H2Se (Fig. 4F). Corresponding oxidative stress responses were determined using a cell permeable fluorescent ROS probe, demonstrating a rapid 20–50% ROS response decrease over the first 3 h following SeNP treatment followed by gradual increase thereafter (Fig. 4G and H).

### 3.4 Selenium induced histone methylation occurs via histone methyltransferase activity

Cellular responses to selenium occur through multiple processes and includes the redox activity of selenoproteins that have incorporated selenocysteine resulting in a protective function against oxidative stress. Having established that SeNP treatment results in intracellular accumulation mostly as Se(IV), we sought to establish whether the similarities in biological effects seen between selenite and SeNP treatments also occurred at the level of transcription by RNA-seq analysis (Supplementary Fig. S2).

Selenium treatment induced the expression of genes encoding selenoproteins (Supplementary Fig. S4). Selenoprotein I, S and T and selenocysteine lyase (SCLY) expression increased in both cell types, with increased SCLY expression suggesting the transformation of selenium to selenocysteine SeCys is likely to be occurring [27]. Other differentially expressed selenoprotein genes included SECI binding protein 2, selenocysteine tRNA synthase and tRNA selenocysteine 1 that were upregulated in SKOV-3, whereas in OVCAR-3 selenoprotein was upregulated but decreased in SKOV-3. Selenium transport selenoproteins SELENBP1 and SEPP1 expressions were not modified through selenium treatment.

**Fig. 5:**
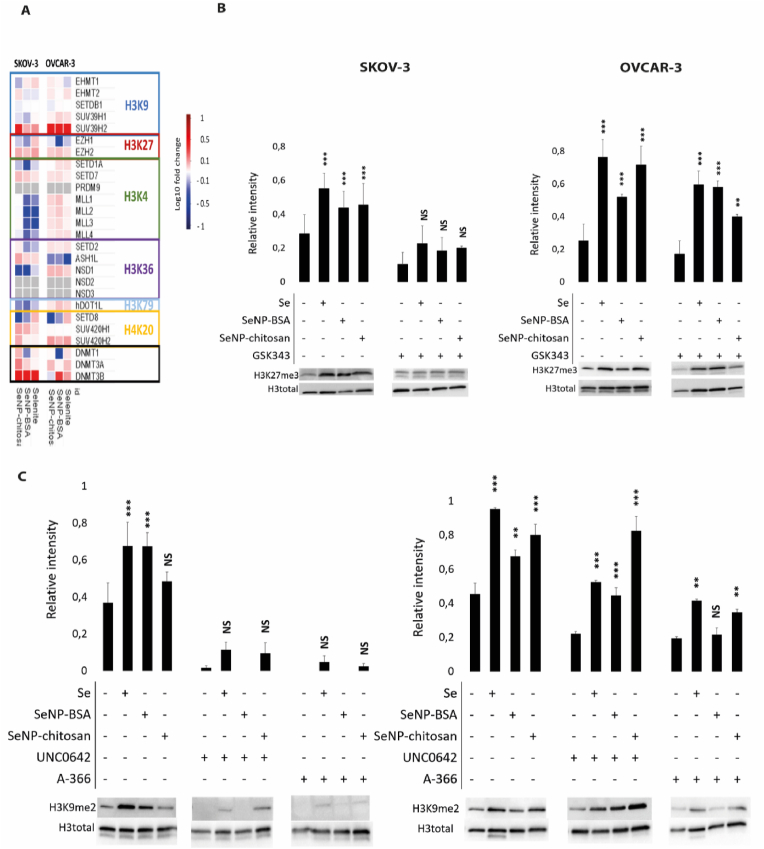
Histone methylation in ovarian cancer cells treated with selenium and epigenetic probes. Methyl transferase expression patterns in SKOV-3 and OVCAR-3 spheroids treated with IC20 concentrations of SeNPs or selenite. (A) Heatmap of methyltransferase gene expression obtained from RNA-seq analysis for SKOV-3 (left), and OVCAR-3 (right) cells after 24 h of SeNP or selenite selenium treatment. SKOV-3 (left) and OVCAR-3 (right) cells were treated with the epigenetic probes (B) GSK343 for 24h or (C) A366 or UNC0642 for 72 h, with all samples subsequently treated with SeNP or selenite for 24 h after the initial HMT inhibitor treatment. (B) Cells were treated for 24 h with the epigenetic probe GSK343, an inhibitor of the H3K27 HMT EZH2 followed by 24 h with selenite or SeNPs. No control probe was available for GSK343. Selenium treatments significantly (p < 0.01) increased H3K27me3 levels by 2 fold in SKOV-3. In OVCAR-3 selenite and SeNP-chitosan increased H3K27me3 levels by 4-fold (p < 0.001) and by 2-fold with SeNP-BSA treatment (p < 0.01). The presence of GSK343 inhibited H3K27me3 methylation in SKOV-3 cells (p > 0.05) but not significantly in OVCAR-3 although expression was decreased. (C) Cells were treated for 24 h with epigenetic probes A-366 or UNC0642, inhibitors of the H3K9 HMT EHMT2/G9a followed by 24 h with selenite or SeNPs. No control probe was available for A-366 or UNC0642. Selenium treatments significantly (p < 0.001) increased H3K9me2 levels by 2 fold in SKOV-3. In OVCAR-3 selenite and SeNP-chitosan increased levels by 2 fold (p < 0.001) and 1.5 fold with SeNP-BSA treatment (p < 0.01). In SKOV-3 UNC0642 and A-366 treatments decreased the levels of H3K9me2 to almost undetectable levels after 24 h and blocked any effect of selenium treatments. In OVCAR-3, UNC0642 and A-366 treatments decreased levels of H3K9me2 (p < 0.05) and reduced the ability of selenium treatments to increase H3K9me2 levels.

Interestingly, SeNPs also induced the expression of several HMT encoding genes including EZH2 which methylates H3K27me3, SETD7 [28] (H3K4me3) and SUV39H2/KMT1B [29,30] (H3K9me3), whilst expression levels of the HMTs EHMT2/G9a and EHMT1/GLP that methylate H3K9me2 were relatively unaffected (Fig. 5). Whilst selenium has been purported to modulate DNA methylation due to changes in DNMT RNA levels following selenium exposure, this is the first observation of any wider involvement of selenium in the regulation of epigenetic mechanisms involving methylation [31,32]. Changes in the expression level of other potential HMTs (as well as lysine demethylases, KDMs) were also observed, although several of the predicted HMTs require further experimental validation to confirm their precise function [33] (Supplementary Fig. S5).

To determine whether the increase in expression of HMTs resulted in an increase in histone methylation, the effects on histone methylation marks were investigated. Following 24 h SeNP treatment we observed an increase in levels of H3K27me3 (Fig. 5B) and H3K9me2 (Fig. 5C), rather than at any one specific methylation mark, thus revealing a novel role for selenium in triggering general histone methylation. Whilst upregulation of SETD7 and EZH2 gene expression by selenium might account for increases in H3K4me3 and H3K27me3 methylation, there were no changes in EHMT2 expression.

To confirm this wider effect on histone methylation, inhibitors blocking the activity of specific HMTs were investigated. Inhibition of the H3K27 methylase EZH2 using GSK343 [34] resulted in a significant decrease in H3K27me3 levels in SKOV-3, demonstrating that the effect of selenium is likely to occur through a process that affects the activity of EZH2 in these cells (Fig. 5B), and also in OVCAR-3 cells although the effect of GSK343 was less pronounced. Finally, two different EHMT2 inhibitors, UNC0642 [35] and A-366 [36], essentially ablated H3K9me2 in SeNP treated SKOV-3 cells (Fig. 5C), with a general decrease in H3K9me2 seen in OVCAR-3 cells. The H3K4me3 HMT PRDM9 was not expressed in the cell lines used and correspondingly inhibition of PRDM9 using MRK740 [37] (or the inactive analogue MRK740 N) did not result in a decrease in H3K4 tri-methylation (Supplementary Fig. S4).

### 3.5 SeNP treatment results in decreased levels of the endogenous HMT inhibitor SAH

**Fig. 6:**
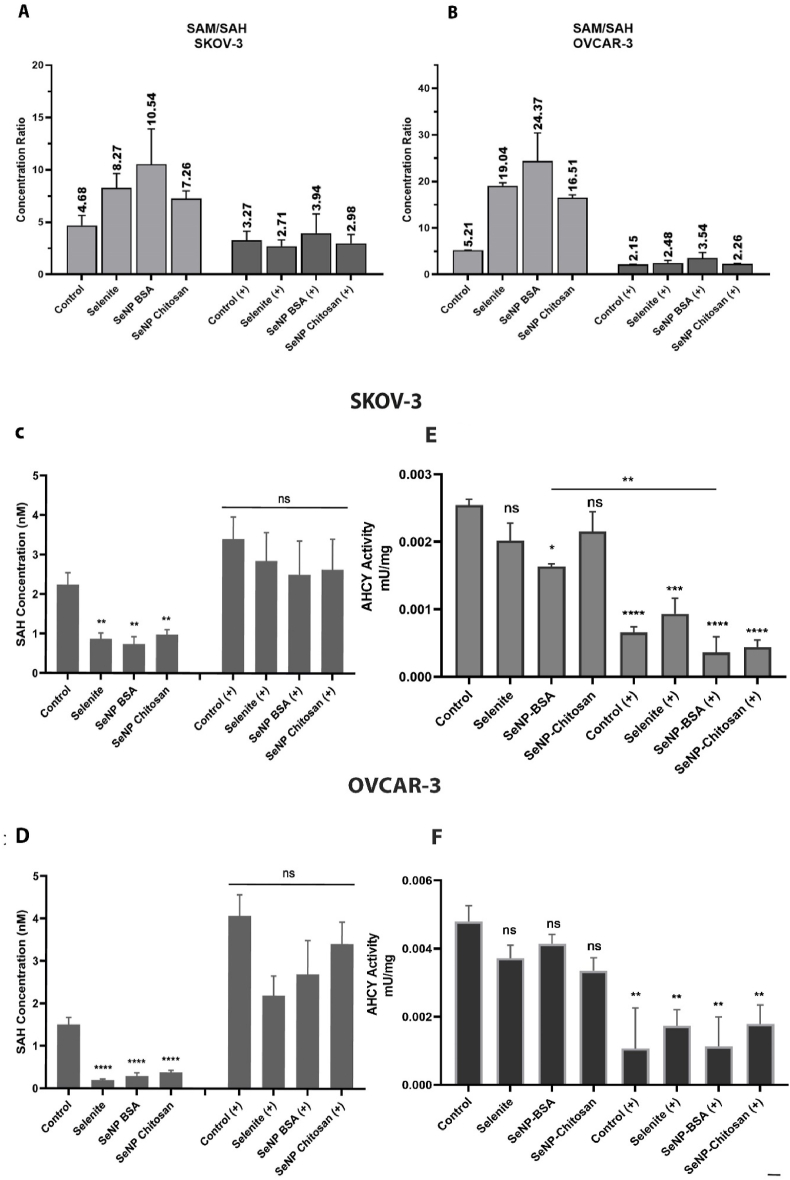
The ratios of SAM to SAH and SAH levels. SAM/SAH ratio was analysed by ELISA and showed an increase in SAM/SAH ratio after selenium treatments in both SKOV-3 (A) and OVCAR-3 (B) with (+) and without AHCY inhibitor. SAH concentrations alter in response to SeNP treatment and inhibition of AHCY. Cells were treated with SeNPs or selenite for 24 h with (+) and without AHCY inhibitor. (C) and (D) SAH levels measured by ELISA, and following all selenium treatments SAH was found to have decreased levels in (C) SKOV-3 and (D) OVCAR-3. This reduction in SAH levels was not observed when cells were treated with the AHCY inhibitor 3-deazaneplanocin at 1 μM for 24 h. (E) and (F) 3-deazaneplanocin effectively inhibits AHCY activity. The concentration of homocysteine in cell extracts was used to determine in vitro AHCY activity, as AHCY hydrolyses SAH to homocysteine and adenosine. Data are mean ± SEM; one-way ANOVA with Tukey multiple comparisons post-hoc analysis; ****p < 0.001, ***p ≤ 0.001, **p < 0.01, *p ≤ 0.05 and ns, not significant vs respective Control (untreated) for each treatment (n = 3 separate experiments). For SeNP-BSA untreated versus treatment was significant (E).

The SAM/SAH ratio is regarded as an indicator of cellular methylation capacity, and an increase in this ratio predicts a higher cellular methylation status. To determine whether SeNP treatment resulted in any alterations of the SAM:SAH ratio, which could influence HKMT activity in addition to the observed increased expression, an ELISA based approach was used and it was observed that SAM:SAH ratios significantly increased after selenium treatment of ovarian cancer cells (Fig. 6A and B). This increase in the SAM:SAH ratio was due to a significant decrease in observed SAH levels following SeNP exposure, which was greater in OVCAR-3 cells compared to SKOV-3 cells (Fig. 6C and D). It appears therefore that the increased activity of different HMTs, and thus increased levels of histone methylation, can be accounted for by a decreased levels in SAH caused by SeNP treatment. As SAH is hydrolyzed exclusively by adenosylhomocysteinase (AHCY) into HCY, we tested whether the decrease in SAH following SeNP treatment was mediated through AHCY hydrolysis using 3-deazaneplanocin, an AHCY inhibitor. First, we established that a significant decrease in AHCY activity could be achieved using 3-deazaneplanocin (Fig. 6E and F). Having blocked AHCY activity the effect of SeNP treatment on SAH levels was re-examined, and in the presence of the AHCY inhibitor revealed a complete restoration of endogenous SAH levels in the presence of SeNP (Fig. 6C and D) and (Supplementary Fig. S5), demonstrating that the ability of SeNP to overcome intrinsic inhibition of histone methylation by SAH accumulation, most likely occurs through enhancing the conversion of SAH to HCY, and not via a direct effect on histone methylating enzymes.

To determine processes that may be regulated by the epigenetic response of ovarian cancer cells to selenium, detailed transcription profiling and gene ontology analysis was undertaken. Specifically, a core set of genes that were regulated by all three selenium treatments (Supplementary Fig. S6A, Table S1) and in both cell types were identified (Supplementary Fig. S6 B, Table S2) and revealed that as well regulating genes involved in oxidative stress including thioredoxin reductase 1 (TXNRD1), selenium also influenced biological pathways and processes including cell adhesion, metal ion binding and protein phosphorylation (Table 1). Consistent with the model for epigenetic regulation, chromatin remodeling was also an enriched term. To understand the relationship between histone methylation and the expression of apoptotic genes, we performed the analysis of publicly available SKOV-3 ChIP-Seq datasets [19]. Using this approach, we identified persistent H3K27me3 peaks surrounding the proximal and/or core promoter regions of both Bcl-2 and p21 (Supplementary Table 3) suggesting a direct link between histone methylation and the regulation of apoptotic genes. We also identified several differentially upregulated genes that have been associated either positively or negatively with ovarian cancer including WIF1 (wnt inhibitory factor 1) the genomic region of which has been shown methylated in association with Se plasma concentration [38] and with ovarian cancer [39], BCL2 binding component 3 (BBC3/PUMA) the regulation of which contributes to the transduction of cell death signals [40] and BBC3/PUMA has been shown to chemosensitize cisplatin resistant ovarian cancer cells [41], mesothelin (MSLN) linked to ovarian cancer progression [42], vascular endothelial growth factor A (VEGFA) associated with the activation of ovarian cancer initiating cells [43], and SUV39H2 (Conlan and Abdulrahmen, unpublished data) demonstrating the wide impact and important considerations for selenium mediated regulating gene expression.

**Table 1:** GO biological process enhanced after 24 h of selenite or SeNP treatment with p-value.

Gene ontology term	P value
oxidoreductase activity	1.5E-1
cell adhesion	5.8E-2
metal ion binding	3.6E-1
protein phosphorylation	4.3E-2
integral component of membrane	7.8E-1
chromatin remodeling	7.7E-2

## 4 Discussion

SeNPs, in contrast to aqueous selenium compounds such as sodium selenite, are reported as well tolerated *in vivo*, and therefore offer a route to unlocking the potential of Se as a therapeutic agent [12]. Indeed, SeNPs had a marked effect on the viability of two distinct ovarian cancer spheroid models evaluated in this study, with SeNPs 2-fold more effective than selenite. Whilst SeNP are composed of Se(0) species, the main selenium species detected within the cells following nanoparticle treatment was Se(IV) suggesting that the effect of SeNPs is likely to be a result of biotransformation into a more available form. Interestingly, whilst the OVCAR-3 form less densely packed spheroids, where nanoparticles might be expected to penetrate more effectively towards cells at the centre of the cell mass [44], the SeNP IC50 was greater than for SKOV-3 which form much more densely packed spheroids. Correspondingly, no difference in penetration depth was observed by TEM indicating that SeNP penetration was similar in both spheroid models. This suggests that the more SeNP tolerant OVCAR-3 spheroids have a different response to SeNP than SKOV-3, and several underlying molecular responses for this were identified.

Histone methylation is associated with apoptosis [45], where the inhibition of H3 methylation at K4 stimulates apoptosis in breast models [46], additionally, H3 methylation at K9 is associated with apoptosis in colon carcinoma cells [47]. SeNP exposure resulted in increased caspase-3 cleavage in SKOV-3 cells suggesting that cell death occurred via apoptosis [48], whereas no apoptosis was induced in OVCAR-3. The cytotoxic effect of SeNP and caspase-3 activation, which was most significant in SKOV3 cells, was related with the general upregulation of ‘Apoptotic metabolic process’ that had the highest enrichment ratio in SKOV3 cells following treatments. We also observed the up-regulation of BCL2 together with corresponding ChIP-Seq peaks associated with H3K27me3 in SKOV3 cells, which together suggest the observed epigenetic response to selenium is likely to contribute to the transduction of cell death and survival signals. Furthermore, in OVCAR-3 cells autophagy was constitutively activated resulting in the intracellular accumulation of SeNPs offering an explanation for the greater resistance of these cells to high levels of selenium. Expression analysis of two apoptotic markers, Bcl-2 and p21 (CDKN1A), further supported the notion that OVCAR-3 is less sensitive to SeNP treatment, as SKOV-3 cells showed a decrease in levels of both apoptosis-related proteins, whereas OVCAR-3 cells showed no significant decrease in either marker following nanoparticle treatment. Assessment of autophagy markers [22] suggested that vacuolar structures observed to contain SeNP were unlikely to be autophagosomes as ATG5 levels and LC3 maturation were not consistently increased following SeNP treatments in either cell line, an observation similar to the vacuolation reported following selenite and GS-Se-SG treatment in HeLa cells [49]. A 40–60 fold increase in the ratio selenodigluthatione (GS-Se-SG) compared to untreated cells was identified using HERFD-XAS. These increases in GSSG/GS-Se-SG were observed in response to SeNP treatment in both OVCAR-3 and SKOV-3 cells. GS-Se-GS is a well-known cellular detoxification pathway for Se and heavy metals [50] and our data suggest it may also interact with the SAH pathway.

In addition to changes in the expression levels of selenoproteins genes, selenium caused alterations in the expression of HMT (and KDM) encoding genes (some of which remain to be experimentally validated [33]). To understand any consequences resulting from changes in the expression of enzymes responsible for affecting epigenetic plasticity we investigated the possibility of selenium inducing epigenetic changes via histone methylation. We demonstrated that both SeNPs and selenite elicited an increase in the levels of H3K27me3 and H3K9me2, rather than at any one specific methylation mark, thus revealing a novel role for selenium in causing general histone methylation. Our study revealed a mechanism whereby selenium-mediated increases in histone methylation occurred through a process involving HMTs, as selenium-induced increases in expression of some HMT genes including EZH2, HMT inhibitors can abrogate this effect. The general increase in histone methylation and inhibition of SeNP mediated methylation by several HMT-specific inhibitors led to the notion that this effect could occur through a ubiquitous process linked to histone methylation. It will be important to determine whether this effect is restricted to cancer cells or whether high levels of selenium result in systemic alterations in histone methylation *in vivo*. Previous studies have shown preferential accumulation of chitosan coated SeNP, functionalised with a targeting peptide, in the liver and tumours in an oesophageal cancer murine model [51]. As the liver is the primary site of selenium accumulation in redox proteins, this is not unexpected. Although a targeting approach was used to demonstrate the effectiveness of SeNP in an *in vivo* model for oesophageal cancer the enhanced permeability and retention effect seen at tumour sites cannot be ruled out for non-targeted SeNP, which may preferentially accumulate in tumours. Furthermore, serum albumin-coated particles such abraxane are recognised by gp60 receptors on endothelial cells forming neovasculature at tumour sites, thus enhancing localised uptake [52].

The methionine metabolic pathway supplies SAM as the methyl-group donor used in histone methylation and generates SAH as a product of the methylation process [53]. SAH functions as an intrinsic HMT inhibitor and its removal from the cellular system due to the presence of high levels of selenium would account for the observed general increases in histone methylation in addition to the observed increase in expression of selected HMTs. Such a decrease in this HMT inhibitor could occur if the metabolic product of SAH, homocysteine (HCY) was actively diverted into the transsulfuration pathway due to the introduction of selenium into this pathway. This would lead to the formation of selenoglutathione (GS-Se-SG) and ultimately selenide (H_2_Se) [54]. We investigated this possibility and observed a significant decrease in SAH following SeNP exposure that was greater in OVCAR-3 cells compared to SKOV-3 cells. SAH levels decreased following SeNP treatment, and this decrease was mediated through AHCY hydrolysis.

**Fig. 7:**
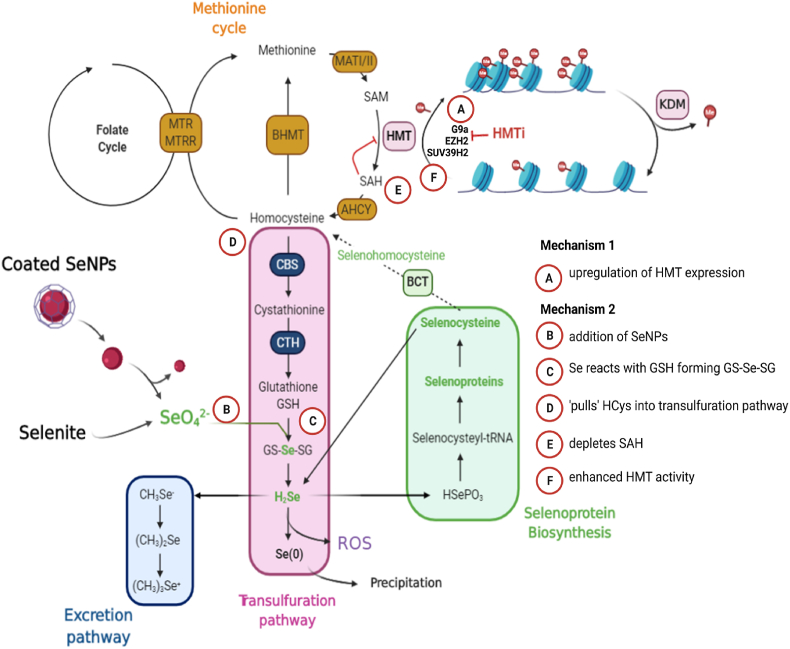
Proposed mechanisms of action for histone methylation induction in ovarian cancer cells. Selenium can influence histone methylation via at least two different mechanisms. **Mechanism 1**. (A) Increasing the expression of HMT genes. **Mechanism 2**. (B) Selenite (SeIV) from the addition of SeNPs or sodium selenite to cancer cells (C) reacts with glutathione GSH forming GS-Se-SG (D) pulling the equilibrium of the methylation SAM-SAH cycle towards homocysteine clearance, (E) depleting endogenous SAH levels thus (F) increasing the activity of HMTs. Histone lysine methyltransferases (HMT), Histone lysine demethylase (KDM), Adenosylhomocysteinase (AHCY), *S*-Adenosylmethionine, (SAM), *S*-Adenosylhomocysteine (SAH), Methionine Synthase Reductase (MTRR), Selenodigluthatione (GS-Se-SG), glutathione GSH. Zeste homolog 2 (EZH2), Euchromatic histone-lysine *N*-methyltransferase 2 (EHMT2/G9a), suppressor of variegation 3–9 homolog 2 (SUV39H2).

Overall we show that SeNP driven increases in histone methylation occur through distinct processes including 1) increasing the activity of HMT due to increasing the levels of expression of the genes encoding these enzymes, the protein products of which can be inactivated by HMT specific inhibitors abrogating the effect of SeNPs and 2) clearance of SAH, possibly due to a ‘pull’ of homocysteine to H_2_Se due to the effects of SeNP on the transsulfuration pathway that resulted in increased levels of selenodiglutathione (Fig. 7). The discovery that selenium, through the activity of HMTs, can modulate histone methylation, a key process in the regulation of global gene expression, highlights the importance of this micro-nutrient. Selenium's pivotal role in redox biology, and its potential applications in cancer [55] and viral [56] therapy, must now also be considered alongside its wider role in the mechanisms of action pertaining to epigenetic processes [32], and for the rationale in advancing anticancer strategies.

## Author contributions

The study was conceived and designed, and critical interpretation of data and drafting of the manuscript were performed by R Steven Conlan, Lewis W Francis and Laurent Charlet. Data collection, data analysis and manuscript drafting were performed by Benoit Toubhans and Nour Al Kafri. Marcos Quintela analysed the RNA sequencing dataset. Se speciation data collection and data analysis were performed by Caroline Bissardon, Sylvain Bohic, and Olivier Proux. Philipp Rathert, Franziska Knodel, Alexandra T. Gourlan and Deyarina Gonzalez contributed to the conception of the project and critical review of the manuscript. The authors read and approved the final manuscript.

## Ethics declarations

The authors declare no competing interests.

## Declaration of competing interest

All authors declare that they have no conflicts of interest.

All authors certify there's no financial/personal interest or belief that could affect their objectivity.

## Data Availability

Data will be made available on request.
