# Supplementary material for: Selenium nanoparticles modulate histone methylation via lysine methyltransferase activity and S-adenosylhomocysteine depletion
Source: Redox Biol. 2023 Feb 23;61:102641. doi: 10.1016/j.redox.2023.102641 (PMC9988660; doi:10.1016/j.redox.2023.102641)
Supplement: Multimedia component 1 [file mmc1.docx]

**Selenium nanoparticles modulate histone methylation via lysine methyltransferase activity and S-adenosylhomocysteine depletion.**

**Authors:** Benoit Toubhans^1, 2,**^, Nour Alkafri^1,**^, Marcos Quintela^1^, David W. James^1^, Caroline Bissardon^3^, Salvatore Gazze^1^, Franziska Knodel^4^, Olivier Proux^5^, Alexandra T. Gourlan^2^, Philipp Rathert^4^, Sylvain Bohic^3,6^, Deyarina Gonzalez^1^, Lewis W. Francis^1^, Laurent Charlet^2^, R. Steven Conlan^1,*^

^1^Swansea University Medical School, Swansea University, Swansea, SA2 8PP, UK

^2^ Université Grenoble Alpes, ISTerre, 38000 Grenoble, France

^3^ Université Grenoble Alpes, INSERM, UA7 STROBE, Synchrotron Radiation for Biomedicine, Grenoble, France

^4^ Department of Biochemistry, Institute of Biochemistry and Technical Biochemistry, University of Stuttgart, D-70550 Stuttgart, Germany

^5^ OSUG, UAR 832 CNRS, Université Grenoble Alpes, 38041, Grenoble, France.

^6^ ESRF, European Synchrotron Radiation Facility, CS 40220, 38043 Grenoble Cedex 9, France

*Corresponding author, mail: [r.s.conlan@swansea.ac.uk](mailto:r.sconlan@swansea.ac.uk)

** contributed equally

# Supplementary information

Supplementary Fig. 1


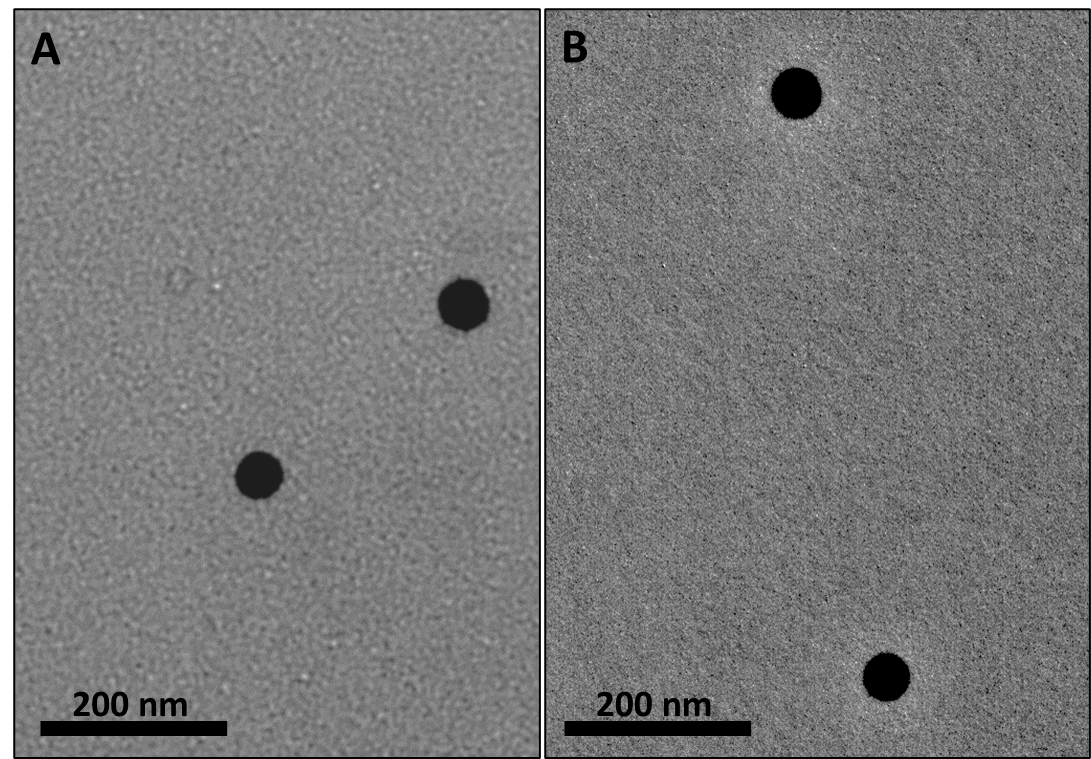


Fig. S1. SeNP characterization and internalization. Representative TEM images of the SeNP-BSA (A) and SeNP-chitosan (B). Transmission Electron Microscopy (TEM) using JEOL 1400-Flash.

Supplementary Fig. 2.


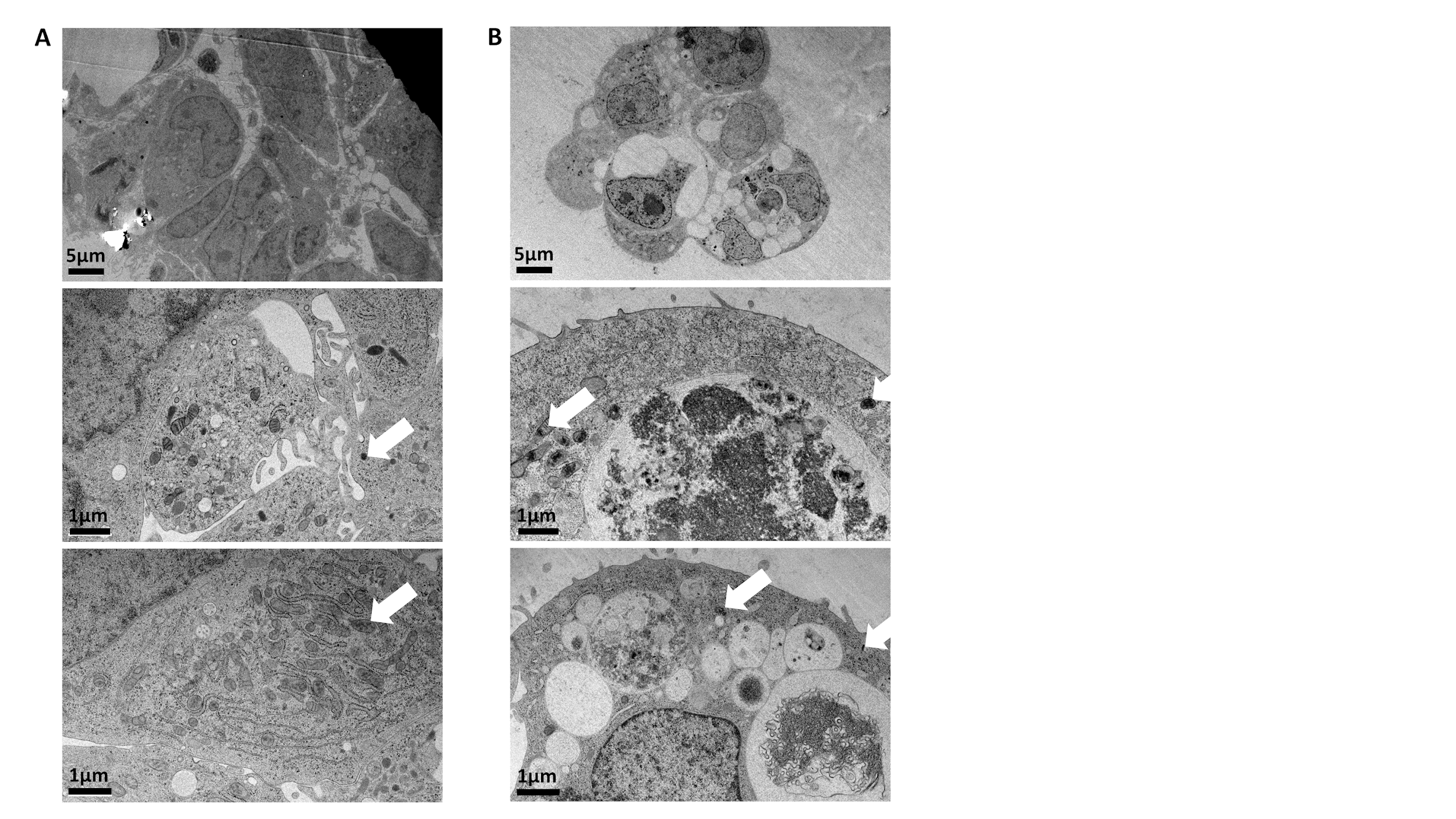


Fig. S2. SeNP-chitosan accumulation in SKOV-3 and OVCAR-3 spheroids.

SKOV-3 (A) and OVCAR-3 (B) cells were grown as 5000 cells spheroids and treated with BSA-SeNPs at sublethal doses for 24 h. TEM preparation was imaged at different magnifications. All images are representative of a minimum 3 biological repeats.

Supplementary Fig. 3


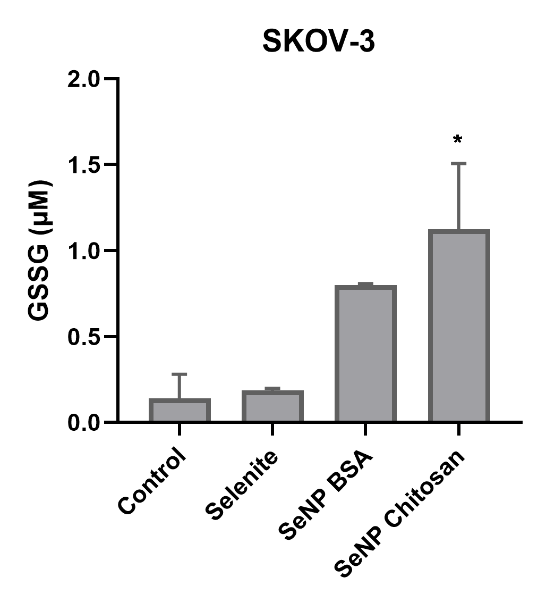


Fig. S3. Quantification of GSSG. SKOV-3 cells were treated with SeNPs for 24 h before the addition of a GSH blocking agent to prevent GSH reduction and subsequently treated with a reducing agent to convert GSSG/GS-Se-SG to GSH prior to quantification via GSH-dependent conversion of luciferin-NT, a GSH probe, to luciferin by a glutathione-S-transferase and showed an increase in GSSG concentrations in SKOV-3 cells after SeNP treatment. Quantitation was made using a standard curve constructed with 0-16 μM of GSH. The limit of detection was 0.5 nM GSSG.

Supplementary Fig. 4


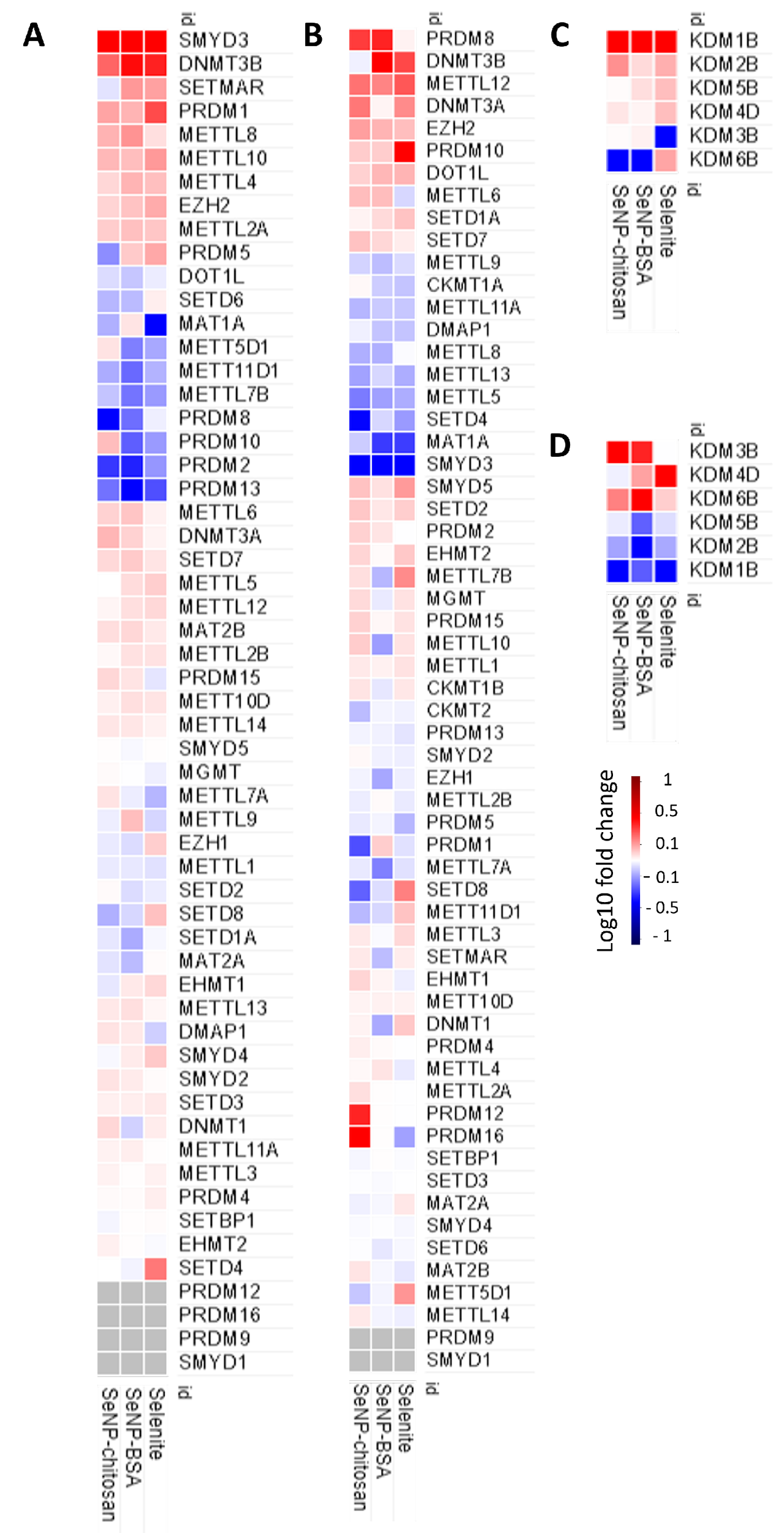


Fig S4. Methyltransferase and lysine demethylase expression patterns in SKOV-3 and OVCAR-3 spheroids treated with sublethal doses of selenium nanoparticles. Heatmaps show log10 expression ratios of methyltransferase (A and B) and lysine demethylase (C&D) genes after 24 h of selenium treatments of SKOV-3 (A and C and OVCAR-3 (B and D) spheroids at sublethal doses.

Supplementary Fig. 5


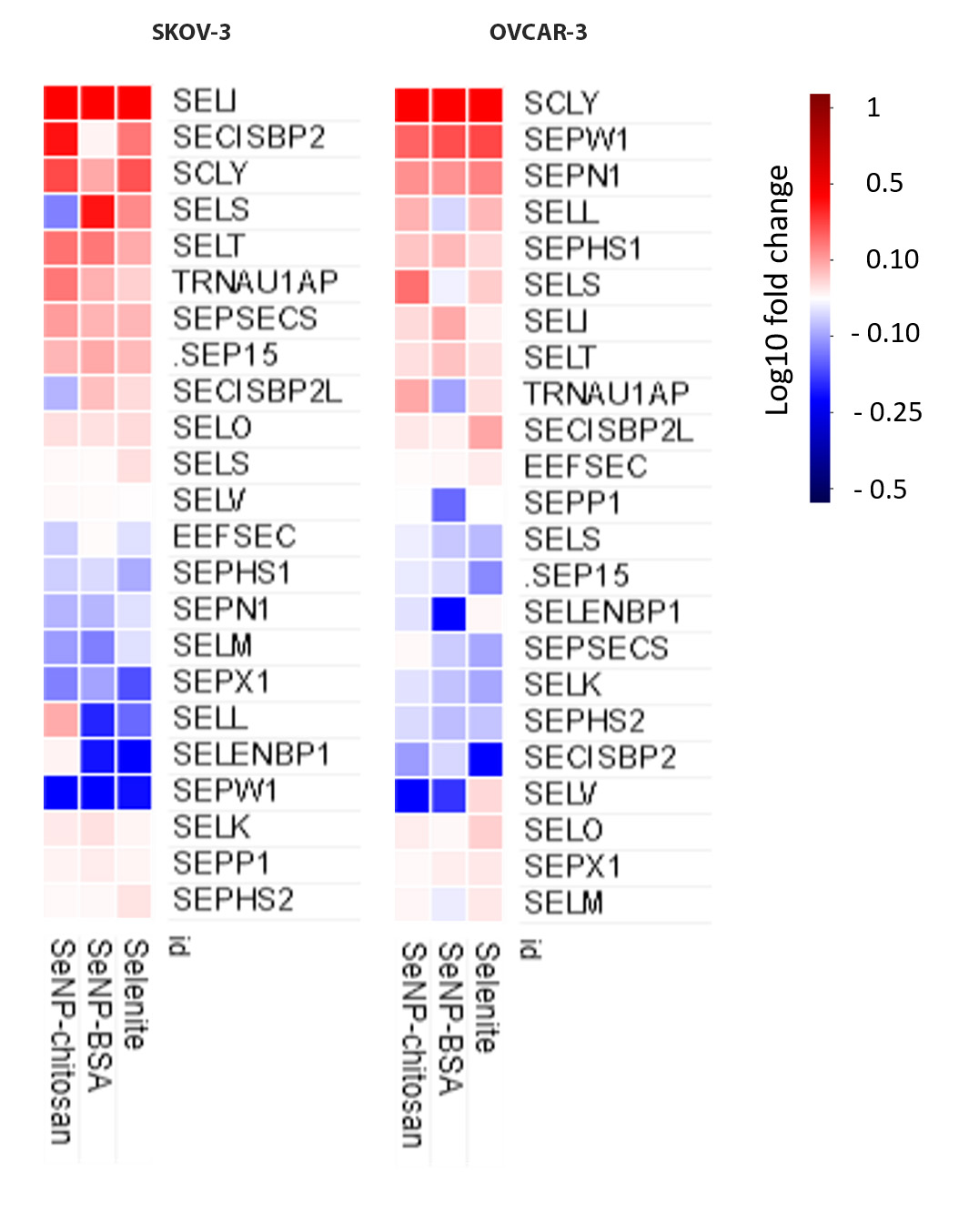


Fig. S5. SKOV-3 and OVCAR-3 cells were treated for 24 h with sublethal doses of selenite or coated selenium nanoparticles. Heatmaps show log10 expression ratios between the selenium treated and control conditions for selenium related proteins.

Supplementary Fig. 6


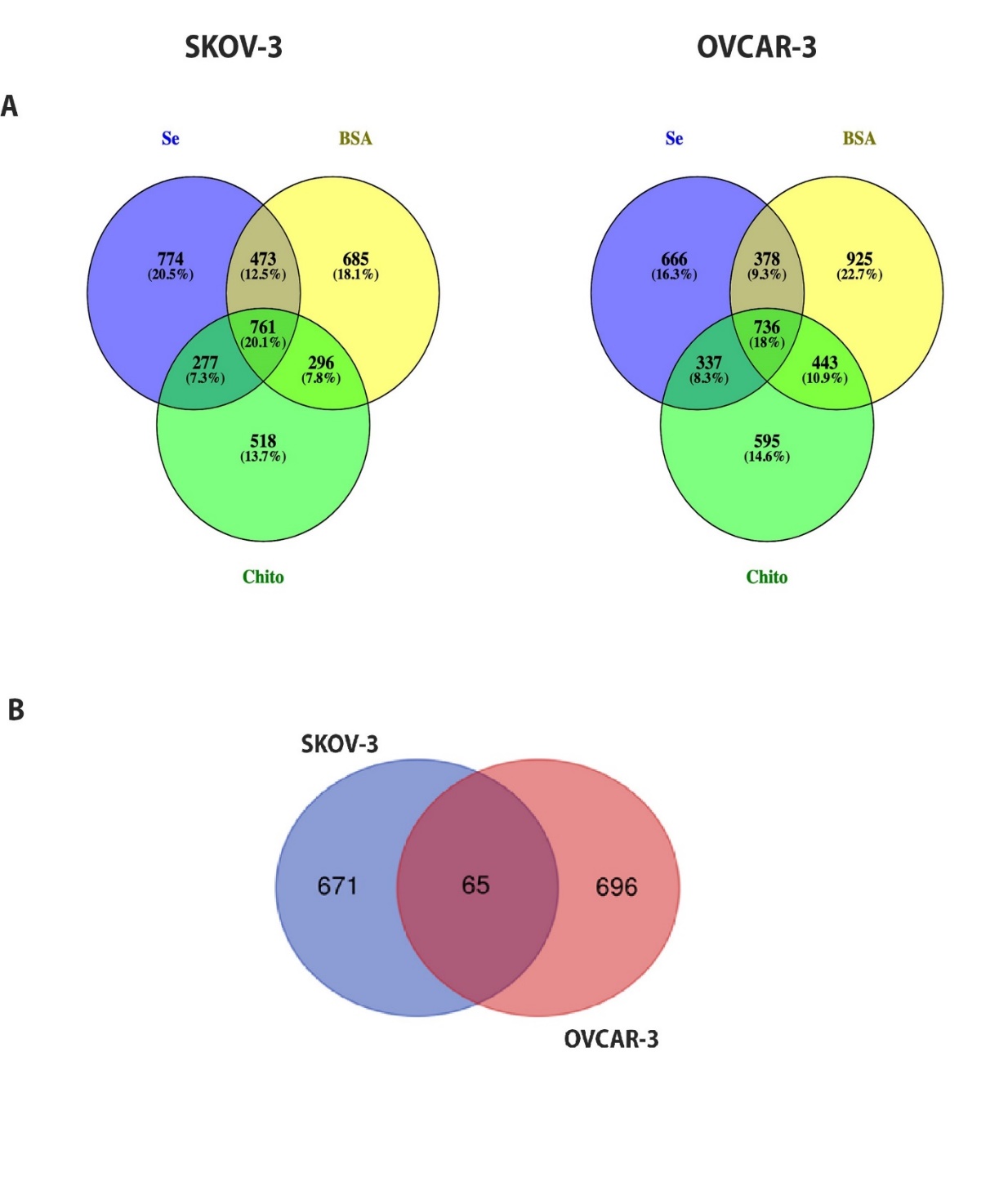


Fig. S6. RNA sequencing data comparison.

(A) Venn diagrams of shared up-regulated genes in SKOV3 and OVCAR3 in responding to the three treatments (Selenite, SeNP-BSA and SeNP-chitosan), and shared genes for the central overlapping regions from OVCAR and SKOV in (B). The 65 genes were used to identify commonly regulated pathways by gene ontology analysis using the DAVID suite of tools.

Supplementary Fig. 7


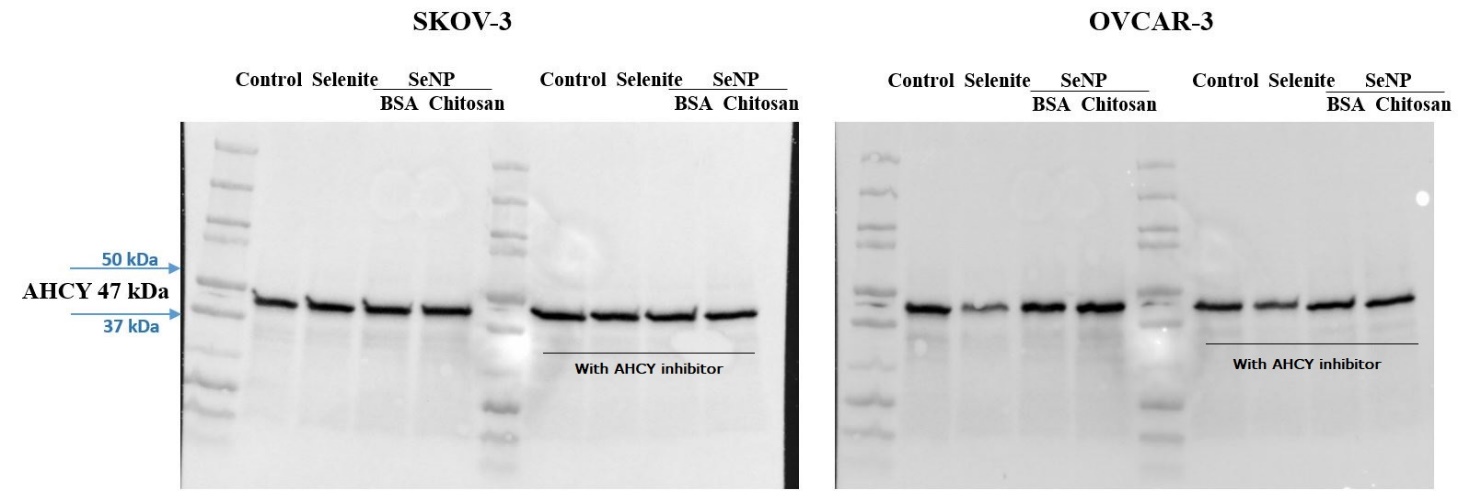


Fig. S7. Protein blot for AHCY levels with and without inhibitor. Cells were treated for 24 h with sublethal doses of SeNPs or selenite with and without AHCY inhibitor at 1 µM. 30 µg of protein was loaded for each sample.

Supplementary Fig. 8


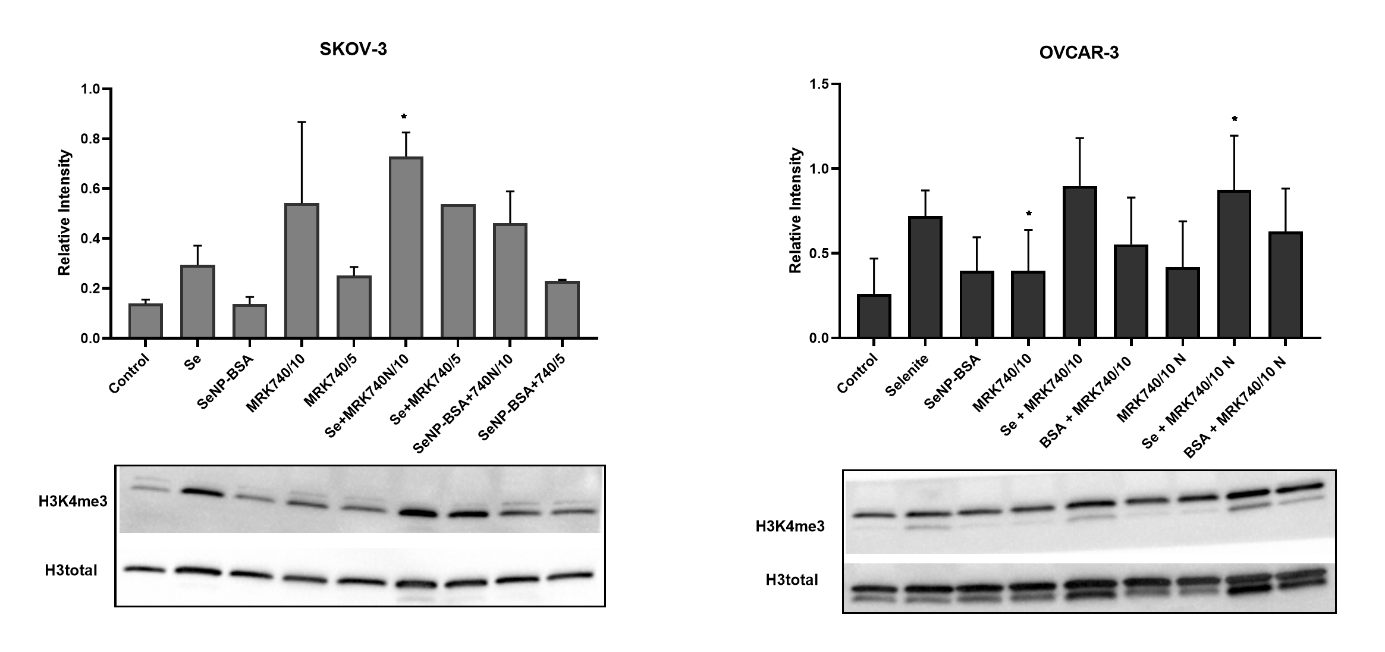


Fig.S8. Histone methylation markers in ovarian cancer cells treated with selenium and epigenetic probes.

SKOV-3 and OVCAR-3 Cells were treated for 72 h with the epigenetic probe MRK740, an inhibitor of the H3K4 HMT PRDM9, followed by 24h with selenite or SeNPs. An inactive probe MRK740N was used as a control. Selenium treatment increased H3K4me3 levels in SKOV-3. In OVCAR-3 selenite increased H3K4me3 levels. The presence of the inhibitor MRK740 did not affect H3K4me3 levels in control or treated samples. Data are mean±SEM; one-way ANOVA with Tukey multiple comparisons post-hoc analysis; **p*<0.05 vs respective Control (untreated) for each treatment (n=3 separate experiments).

Supplementary Table 1**:** GO biological process (PANTHER terms) enhanced before and after 24 h of selenite or SeNP treatment with Fold Enrichment and False Discovery Rate (FDR).

|  | **Gene over-representation pathways** | **Enrichment Ratio** | **FDR** |
| --- | --- | --- | --- |
| **SKOV-3** | Apoptotic metabolic process  Oxidative stress response  Reactive oxygen species pathway  NRF2 pathway  Biological oxidations  Cellular response to stress | 6.78  5.68  4.38  3.40  2.79  1.60 | 2.57^E^-03  1.02^E^-02  1.90^E^-02  1.18^E^-03  6.41^E^-03  2.57^E^-03 |
| **OVCAR-3** | PDGFR-beta pathway  Regulation of calcium ion transport  Oxidative stress response  Interferon gamma response  MAPK Signaling pathway  Regulation of cell death  Regulation of intracellular signal transduction | 4.88  3.28  2.66  2.42  2.30  1.69  1.61 | 4.02^E^-02  5.01^E^-04  7.22^E^-02  5.78^E^-03  3.36^E^-02  5.01^E^-04  1.13^E^-03 |

Supplementary Table 2. Genes upregulated >1.5 fold in response to selenite and SeNP-BSA and SeNP-chitosan in both SKOV3 and OVCAR3 cells.

| Gene | Function |
| --- | --- |
| DHRS12 | dehydrogenase/reductase 12(DHRS12) |
| UCN | urocortin(UCN) |
| SUV39H2 | SUV39H2 histone lysine methyltransferase(SUV39H2) |
| PCDHGB7 | protocadherin gamma subfamily B, 7(PCDHGB7) |
| CD82 | CD82 molecule(CD82) |
| MSLN | mesothelin(MSLN) |
| LARP1B | La ribonucleoprotein 1B(LARP1B) |
| BBC3 | BCL2 binding component 3(BBC3) |
| TRIM9 | tripartite motif containing 9(TRIM9) |
| TMEM102 | transmembrane protein 102(TMEM102) |
| RGS3 | regulator of G protein signaling 3(RGS3) |
| PRX | periaxin(PRX) |
| SCMH1 | Scm polycomb group protein homolog 1(SCMH1) |
| HYAL3 | hyaluronidase 3(HYAL3) |
| DIP2A | disco interacting protein 2 homolog A(DIP2A) |
| ZNF442 | zinc finger protein 442(ZNF442) |
| PRKCG | protein kinase C gamma(PRKCG) |
| HSD11B1L | hydroxysteroid 11-beta dehydrogenase 1 like(HSD11B1L) |
| CCNB1IP1 | cyclin B1 interacting protein 1(CCNB1IP1) |
| BACE2 | beta-secretase 2(BACE2) |
| PPM1B | protein phosphatase, Mg2+/Mn2+ dependent 1B(PPM1B) |
| ZNF92 | zinc finger protein 92(ZNF92) |
| PDE5A | phosphodiesterase 5A(PDE5A) |
| ERG | ETS transcription factor ERG(ERG) |
| PPP1R12B | protein phosphatase 1 regulatory subunit 12B(PPP1R12B) |
| DEPDC5 | DEP domain containing 5, GATOR1 subcomplex subunit(DEPDC5) |
| FBLIM1 | filamin binding LIM protein 1(FBLIM1) |
| CCDC24 | coiled-coil domain containing 24(CCDC24) |
| SLC41A3 | solute carrier family 41 member 3(SLC41A3) |
| DBF4B | DBF4 zinc finger B(DBF4B) |
| PRDX5 | peroxiredoxin 5(PRDX5) |
| RECQL5 | RecQ like helicase 5(RECQL5) |
| MAP2 | microtubule associated protein 2(MAP2) |
| GRK4 | G protein-coupled receptor kinase 4(GRK4) |
| WIF1 | WNT inhibitory factor 1(WIF1) |
| TMEM68 | transmembrane protein 68(TMEM68) |
| MIER1 | MIER1 transcriptional regulator(MIER1) |
| SH3BP5 | SH3 domain binding protein 5(SH3BP5) |
| SEC23B | SEC23 homolog B, COPII coat complex component(SEC23B) |
| UGT1A6 | UDP glucuronosyltransferase family 1 member A6(UGT1A6) |
| B3GALNT1 | beta-1,3-N-acetylgalactosaminyltransferase 1 (globoside blood group)(B3GALNT1) |
| BDNF | brain derived neurotrophic factor(BDNF) |
| ODF2 | outer dense fiber of sperm tails 2(ODF2) |
| LVRN | laeverin(LVRN) |
| TXNRD1 | thioredoxin reductase 1(TXNRD1) |
| KCNJ14 | potassium inwardly rectifying channel subfamily J member 14(KCNJ14) |
| HPS1 | HPS1 biogenesis of lysosomal organelles complex 3 subunit 1(HPS1) |
| AKR1C2 | aldo-keto reductase family 1 member C2(AKR1C2) |
| DDHD1 | DDHD domain containing 1(DDHD1) |
| TRPV1 | transient receptor potential cation channel subfamily V member 1(TRPV1) |
| BMX | BMX non-receptor tyrosine kinase(BMX) |
| VEGFA | vascular endothelial growth factor A(VEGFA) |
| TNRC6C | trinucleotide repeat containing adaptor 6C(TNRC6C) |
| NFASC | neurofascin(NFASC) |
| PTK7 | protein tyrosine kinase 7 (inactive)(PTK7) |
| FAM131A | family with sequence similarity 131 member A(FAM131A) |
| PPP1R14D | protein phosphatase 1 regulatory inhibitor subunit 14D(PPP1R14D) |

Supplementary Table 3. Table summarising genomic position and average peak score (x̄) of ChIP-Seq peaks associated with H3K27me3 in SKOV3 cells.

| **Gene** | **H3K27me3** | |
| --- | --- | --- |
|  | **Position** | **x̄ Peak score** |
| **CDKN1A/p21** | Core promoter | 52 |
| **BCL2** | Proximal promoter | 38 |
| **UCN** | Core promoter  Gene body | 215  104 |
| **TRIM9** | Proximal promoter  Core promoter  Gene body | 42  65  28 |
| **WIF1** | Proximal promoter  Core promoter | 155  268 |
| **PRKCG** | Core promoter | 178 |
